# A Hybrid Landslide Displacement Prediction Method Based on CEEMD and DTW-ACO-SVR—Cases Studied in the Three Gorges Reservoir Area

**DOI:** 10.3390/s20154287

**Published:** 2020-07-31

**Authors:** Junrong Zhang, Huiming Tang, Tao Wen, Junwei Ma, Qinwen Tan, Ding Xia, Xiao Liu, Yongquan Zhang

**Affiliations:** 1 Faculty of Engineering, China University of Geosciences, Wuhan 430074, Hubei, China; zjr@cug.edu.cn (J.Z.); tanghm@cug.edu.cn (H.T.); tanqinwen@cug.edu.cn (Q.T.); cug_xia@cug.edu.cn (D.X.); 2 School of Geosciences, Yangtze University, Wuhan 430100, Hubei, China; wentao200840@yangtzeu.edu.cn; 3 Three Gorges Research Center for Geohazards of Ministry of Education, China University of Geosciences, Wuhan 430074, Hubei, China; majw@cug.edu.cn (J.M.); liuxiao@cug.edu.cn (X.L.)

**Keywords:** landslide displacement, hybrid prediction model, complete ensemble empirical mode decomposition (CEEMD), dynamic time warping (DTW), ant colony optimization (ACO), support vector regression (SVR)

## Abstract

Accurately predicting the surface displacement of the landslide is important and necessary. However, most of the existing research has ignored the frequency component of inducing factors and how it affects the landslide deformation. Therefore, a hybrid displacement prediction model based on time series theory and various intelligent algorithms was proposed in this paper to study the effect of frequency components. Firstly, the monitoring displacement of landslide from the Three Gorges Reservoir area (TGRA) was decomposed into the trend and periodic components by complete ensemble empirical mode decomposition (CEEMD). The trend component can be predicted by the least square method. Then, time series of inducing factors like rainfall and reservoir level was reconstructed into high frequency components and low frequency components with CEEMD and *t*-test, respectively. The dominant factors were selected by the method of dynamic time warping (DTW) from the frequency components and other common factors (e.g., current monthly rainfall). Finally, the ant colony optimization-based support vector machine regression (ACO-SVR) is utilized for prediction purposes in the TGRA. The results demonstrate that after considering the frequency components of landslide-induced factors, the accuracy of the displacement prediction model based on ACO-SVR is better than that of other models based on SVR and GA-SVR.

## 1. Introduction

As one of the most widely distributed and frequently occurring geological disasters in nature, landslides pose a greater threat to the environment, natural resources, hydraulic engineering, etc. [1]. A landslide can be regarded as a nonlinear dynamic system, which is affected by external factors such as rainfall, reservoir water level, and groundwater [2]. It is reported that plenty of old landslides were reactivated by the periodic reservoir level fluctuation and rainfall since the first impound of the Three Gorges Reservoir (TGR) in 2003 [3,4]. The surface displacement of the landslide is one of the important information generated during the landslide deformation process and is of great significance to predict the evolution law and development trend of landslides according to the analysis of it [5,6,7]. Accurately predicting the surface displacement of the landslide is important and necessary for mastering the evolution stages of landslide and realizing the accurate early warning [8,9].

The support vector machine (SVM) proposed by Vapnik [10], as a popular machine learning method that offers solutions for both classification and regression problems, has been widely used in snow avalanche hazard prediction [11], earth fissure hazard prediction [12], landslide displacement prediction [13,14], etc. The support vector regression (SVR) algorithm is the regression method of SVM, which has many applications in the prediction of time series combined with time-series theory [15]. The landslide displacement time series is generally regarded as the superposition of the trend component and the periodic component and is predicted separately by the least square method and SVR model. However, the dominant limitation of the SVR model, that is, the penalty parameter C and the kernel function parameter g is difficult to determine, and its accuracy is directly related to the model’s predictive ability [16,17,18,19,20]. Thus, the selection of an optimization algorithm for the SVR model plays a very important role [21]. Miao et al. [22] compared the prediction results of the genetic algorithm (GA)-SVR, grid search (GS)-SVR, and particle swarm optimization (PSO)-SVR and found that GA-SVR gave the best results. Zhou et al. [23] developed displacement prediction models based on the analysis of time series data and a PSO-SVR model and obtained accurate results. Cai et al. [24] and Wen et al. [25] proposed a least-squares support vector machine (LSSVM) approach with multiple factors and a genetic algorithm (GA). Bui et al. [26] establish a hybrid intelligent method for the spatial prediction of rainfall-induced landslides by combining LSSVM with artificial bee colony (ABC) optimization.

However, these optimization algorithms all have the disadvantages of falling into a local optimum easily in high-dimensional space and have a low convergence rate in the iterative process. Therefore, ant colony optimization (ACO) as a new biological evolution simulating method, which has the advantages of parallel computing, positive feedback search, and good adaptability can be used to avoid this issue [27,28]. The ACO algorithm is easy to realize. Meng et al. [29] proposed a pricing model with ACO-based SVR, utilized ACO to increase the generalization performance. Hong et al. [30] and Zhou et al. [31] used the ACO-SVR model for forecasting purposes, and find that ACO can automatically determine the optimal parameters of SVR with higher predictive accuracy and generalization ability simultaneously. Overall, the existing research has proved that the ACO can facilitate the selection of optimal parameters for SVR, which has great potential in landslide displacement prediction [32].

The choice of input variables for the SVR model is vital [33]. Even though the inducing factors like rainfall show a significant seasonal characteristic, most of the existing research has ignored the frequency component of it and how it affects the deformation of the landslide. Zhang et al. [34] pointed out that the IMFs of empirical mode decomposition (EMD) are extracted from high to low frequencies according to frequencies. Deng et al. [35] used the ensemble empirical mode decomposition (EEMD) and *t*-test methods to extract the frequency components of the inducing factors, based on which, a good prediction effect was obtained. Thus, as an optimized EMD, complete ensemble empirical mode decomposition (CEEMD) combined with a *t*-test can highlight the fluctuations in a time series such as high frequency and low frequency components of rainfall and reservoir water levels. High frequency components usually reflect the characteristics of high intensity and frequency of influencing factors, such as multiple heavy rainfalls in a short time, leading to landslide deformation. The low frequency component reflects the factors related to the continuous creep movement under gravitational loads. Additionally, since the periodic component is a complex curve, which affected mostly by external inducing factors like the rainfall or reservoir level, it is critical to select the appropriate inducing factors as the explanatory variable for the prediction of the periodic component according to its generation mechanism [36,37]. At present, the selection methods of input variables mainly include gray relational analysis (GRA) [38], mean influence value (MIV) [39], maximal information coefficient (MIC) [7], etc. Dynamic time warping (DTW) as a common time series similarity measurement method is widely used in the time-varying data sequence match since proposed by Berndt due to its simple concept and robust [40]. It can be utilized in the selection of dominant inducing factors for the prediction of landslides.

By considering the frequency components of inducing factors, optimized parameter selection methods and application of ACO optimization algorithm, this study presents a hybrid prediction method consisting of the CEEMD, the DTW, and the ACO-SVR model to improve the accuracy of the SVR-based prediction model. The monitoring displacement data of landslide were first decomposed into the trend and periodic component by the CEEMD method. The trend component can be fitted and predicted by the least square method. Then, the inducing factors like rainfall and reservoir levels time series were reconstructed into high frequency components, low frequency components, and others with CEEMD and a *t*-test, respectively. The dominant factors were selected by the method of DTW from the inducing factor group. Finally, the ACO algorithms were used to determine the optimal C and g for the SVR model, which is then trained to predict the periodic component of the landslide displacements. Baishuihe landslide and Shuping landslide from the Three Gorges Reservoir area (TGRA) were studied to verify the effectiveness of the proposed hybrid method. The application of this method demonstrates the preferable effect of it and can be applied to the prediction of other creep landslides affected by the seasonal rainfall and reservoir water level.

## 2. Proposed Methodology

### 2.1. Time Series Theory of Displacement Data

The deformation of the landslide is mainly affected by the internal and external inducing factors. Internal inducing factors like geological structures, topography, landforms, etc., controls the displacement performance as an approximately monotonically increasing function over time, reflecting the general trend of the cumulative displacement change of the landslide. The external inducing factors like rainfall, reservoir level changes, etc., controls displacement as an approximately periodic function over time. Besides, there are random displacements caused by random factors such as wind load and traffic load [22]. Therefore, according to the time series addition model, the landslide displacement curve can be decomposed as follows:

(1) X(t)=ζ(t)+η(t)+μ(t)

where, X(t) is the observed displacement data of time t, ζ(t) is the trend component of time t, η(t) is the periodic component of time t, μ(t) is the random component of time t.

Due to the limitation of current monitoring methods, the inducing factors of random components have not been well obtained and have been ignored in this article. Formula (1) can be simplified into:

(2) X(t)=ζ(t)+η(t)

### 2.2. Complete Ensemble Empirical Mode Decomposition

Complete ensemble empirical mode decomposition (CEEMD), as a modified algorithm of EMD and EEMD, is widely used for time-frequency analysis adapted to non-stationary signals due to its high computing efficiency [41]. The realization of CEEMD is as follows:

(1) Adding n sets of white noise sequences to the original signal in the form of positive and negative pairs, then 2n set signals are generated.

(3) $$\left[\begin{array}{cc} 1 & 1\\ 1 & -1 \end{array}\right]\left[\begin{array}{c} S\\ N \end{array}\right]=\left[\begin{array}{c} Z_{1}\\ Z_{2} \end{array}\right]$$

where $Z_{1}$ and $Z_{2}$ are time series after adding positive and negative white noise, respectively; N is the added white noise; S is the original time series.

(2) The EMD algorithm is used to decompose each set of signals, and each set of the signal gets a set of m−1 intrinsic mode function (IMF) components and a residue.

(3) The corresponding IMF components and the residue are averaged as the final decomposition result.

(4) $$c_{j}=\frac{1}{2}\overset{2n}{\underset{i=1}{\sum }}c_{ij}+\frac{1}{2n}\overset{2n}{\underset{i=1}{\sum }}c_{im}$$

where $c_{j}$ is the IMF component after decomposition, 1≪j≪m, $c_{m}$ is the residue; $c_{ij}$ is the *j*th IMF component of *i*th signal; and n denotes the number of white noises, 1≪i≪n.

### 2.3. Dynamic Time Warping

Dynamic time warping (DTW) is the algorithm originally used in the field of speech recognition. It is a non-linear warping technique combining time warping and distance measurement calculation and has been widely used for calculating the similarity between time series [42,43]. The dynamic time warping distance (DTWD) can be calculated as follows:

(1) Set time series $T=\left\{T_{1},\;T_{2},\ldots ,\;T_{n},\;\ldots ,\;T_{N}\right\}$ as the test sample sequence; set time series $R=\left\{R_{1},\;R_{2},\ldots ,\;R_{m},\;\ldots ,\;R_{M}\right\}$ as a reference sample sequence. Set matrix $A_{M\times N}={\left(a_{mn}\right)}_{M\times N}$ as the distance matrix of T and R, then, $a_{mn}={\left(T_{n}-R_{m}\right)}^{2}$

(2) In the distance matrix, set $W=w_{1},w_{2},\ldots ,w_{k}$ as the dynamic time warping path (DTWP) of the test sample sequence and reference sample sequence. Where, $w_{k}={\left(a_{mn}\right)}_{k}$ is *k*th elements of the DTWP. The path W needs to meet the following conditions:

(5) $$\{\begin{array}{l} max\left\{M,N\right\}\leq K\leq M+N-1\\ w_{1}=a_{11},w_{k}=a_{MN}\\ if\;w_{k}=a_{mn},\;w_{k+1}=a_{mn},\;then,\;0\leq m^{\prime }-m\leq 1\;and\;0\leq n^{\prime }-n\leq 1 \end{array}$$

(3) DTWP is not unique, so the DTWP with the minimum value of $\sqrt{\overset{K}{\underset{m=1}{\sum }}w_{m}}$ is the best and the corresponding distance is the dynamic time warping distance. Set DTW(T,R) as the dynamic time warping distance between test sample sequence T and reference sample sequence R, then:

(6) $$DTW\left(T,R\right)=\min(\sqrt{\overset{K}{\underset{m=1}{\sum }}w_{m}})$$

Set accumulate distance of test sample sequence $\;T_{m}$ and reference sample sequence $R_{n}$ as $L_{mn}$, so

(7) $$L_{mn}=min\left[L\left(m-1,n-1\right),L\left(m-1,n\right),L\left(m,n-1\right)\right]+a_{mn}$$

where $L_{1,1}=a_{11}$, so  DTW(T,R)=L(M,N).

By calculating the DTWD between the inducing factors and the periodic component, the smaller the calculation result is, the higher correlation the inducing factor is with the periodic component. Consequently, the inducing factor with the smallest result is selected as the input variable for the SVR model.

### 2.4. Support Vector Regression Model

The support vector regression (SVR) algorithm is widely used for small samples training and has advantages of being a simple process, having accurate prediction results, and having strong robustness [22,44]. It divides sample data into the training sample and test sample, taking the training sample as the input vector, then maps it to higher dimensional feature space, and trains it. Next, the best fitting effect is obtained in the space of the optimal decision function model, and the training sample is used to validate the analytical model results [15]. However, due to a lack of theoretical methods available to determine the penalty factor and the kernel function parameter (C, g), the approach for selecting C and g must be further studied [7,14]. In this paper, ACO was adopted to obtain the optimal parameters for the SVR model, and its performance was compared to the original SVR model and GA-SVR model.

### 2.5. Ant Colony Optimization

As a general-purpose stochastic optimization algorithm, the ACO mimics the behavior of ants in finding the shortest paths from the colony to food. In this process, ants will leave pheromones on the paths they pass, and the ants followed will then choose paths based on the pheromone’s intensity. When reaching an intersection that has not been passed, ants will randomly select a path and released pheromones, and the amount of pheromone is inversely proportional to the length of the path. Over time, the pheromone on the shorter path will continue to increase, while the pheromone on the other longer paths will gradually decrease or disappear, and eventually, the ant colony will find a suitable optimal path. To imitate this, the artificial in ACO ants performs a mobile search through positive feedback of volatility accumulation of pheromones to select the optimal path, which can avoid falling into the local optimum. Therefore, the key of the ACO lies in the movement rules and pheromone update [45,46,47,48]. The detailed steps of ACO-SVR are as follows:

Step 1: initialize parameters and variables of the proposed algorithm by randomly assigning a set of parameters (C, g) to each artificial ant. Therefore, the corresponding error model can be obtained by training the training-data through SVR:

(8) $$Error\left(i\right)=V_{train}-V_{real}$$

where $V_{train}$ are the results of the SVR model, $V_{real}$ is the value of the training data. Then, the pheromone of ant in i position can be obtained according to the error value predicted by the above error model:

(9) $$T_{0}\left(i\right)=\alpha^{-Error\left(i\right)}$$

where α is set to 3, so the smaller the error value, the larger the pheromone.

Step 2: Define the artificial ant’s transfer probability. The transfer probability of each artificial ant can be obtained based on the value of the pheromone:

(10) $$p\left(i\right)=\frac{e^{T_{0}\left(BestIndex\right)-T_{0}\left(i\right)}}{e^{T_{0}\left(BestIndex\right)}}$$

where BestIndex is the artificial ant with the highest value of the pheromone.

Step 3: Define the evaporation coefficient ρ. To avoid local optimum, this study made the evaporation coefficient relatively small at the beginning of the local search and gradually increased with times of iterations. The evaporation coefficient ρ is defined as:

(11) $$\rho =K\times \frac{log\left(9\right)\times Echo}{e^{Echo_{max}}}$$

where K=0.1, Echo is the times of iterations and $Echo_{max}$ is the max times of iterations.

Step 4: In each iteration, a dynamic global transfer factor $P_{0}$ is established based on the value of the pheromone. After setting the number of artificial ants as *m*, calculate the value of $e^{-T_{0}\left(i\right)},\;i=1,2,\ldots ,m$, then sort the results in ascending order to get a new sequence $T_{i}\left(j\right),\;j=1,2,\ldots ,m$. When $Echo<\left(\frac{Echo_{max}}{2}\right)$, $P_{0}=t_{1}\left(k\right)$, $k=\frac{2}{3}m$; otherwise, $P_{0}=t_{1}\left(k\right)$, $k=\frac{1}{5}m$.

Use the $P_{0}$ as a criterion, perform the local search when transfer probability $p\left(i\right)<P_{0}$, otherwise, perform a global search. At the beginning of the search, most of the ants will perform the local search for a better solution. After that, most of the ants perform the global search to avoid falling into a local optimum and obtain a globally optimal solution.

Step 5: Update pheromone based on the C and g obtained from ACO, the update rules are as follows:

(12) T_0 (i)=(1−ρ)×T_0 (i)+∆t(i)

Step 6: Save the optimal solution in each iteration. Keep the ant with the largest pheromone in each iteration and calculate the error value according to the error model, return to step 1, and perform the iteration cycle.

Step 7: The global optimal solution obtained. When the iteration is accomplished, the best ant with the best combination of C and g is obtained based on the error value.

Step 8: Calculate the corresponding results of SVR according to the best combination of C and g.

### 2.6. Procedure of the Proposed Hybrid Algorithm

**Figure 1:**
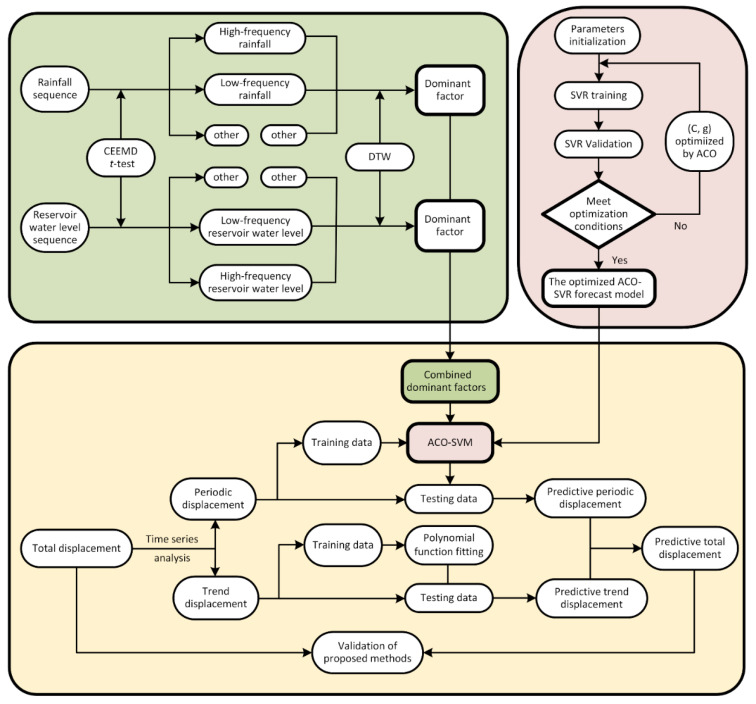
Schematic diagram of the proposed hybrid prediction method.

The schematic diagram of the proposed hybrid model is present in Figure 1. First, CEEMD was used to decompose the cumulative displacement time series into a trend and periodic components based on time series theory, in which, the trend component was then fitted and predicted by a polynomial function. CEEMD and *t*-test methods were used to reconstruct the time series data of rainfall and reservoir level into high- and low frequency components. The two dominant influential factors with the smallest DTWD were selected through DTW for the prediction of the periodic component. Finally, the ACO algorithm was used to determine the optimal *C* and *g* for the SVR model, which was then trained to predict the periodic component of the landslide displacements. The predicted result of the cumulative displacement was the sum of the predicted trend component and the predicted periodic component.

## 3. Cases Study

The Three Gorges Dam (TGD) is now the world’s largest water conservancy project, causing numerous landslides and other geological hazards in this area. The banks of mainstream and tributaries in TGRA have a total length of 5300 km; many giant ancient landslides are distributed on both sides of the TGRA. Since the reservoir began to impound water, a series of ancient landslides were reactivated and deformed, meanwhile, new landslides were generated. For example, when the reservoir water level reached 135 m in 2003, more than 200 landslides in the reservoir area began to strongly deform [1].

**Figure 2:**
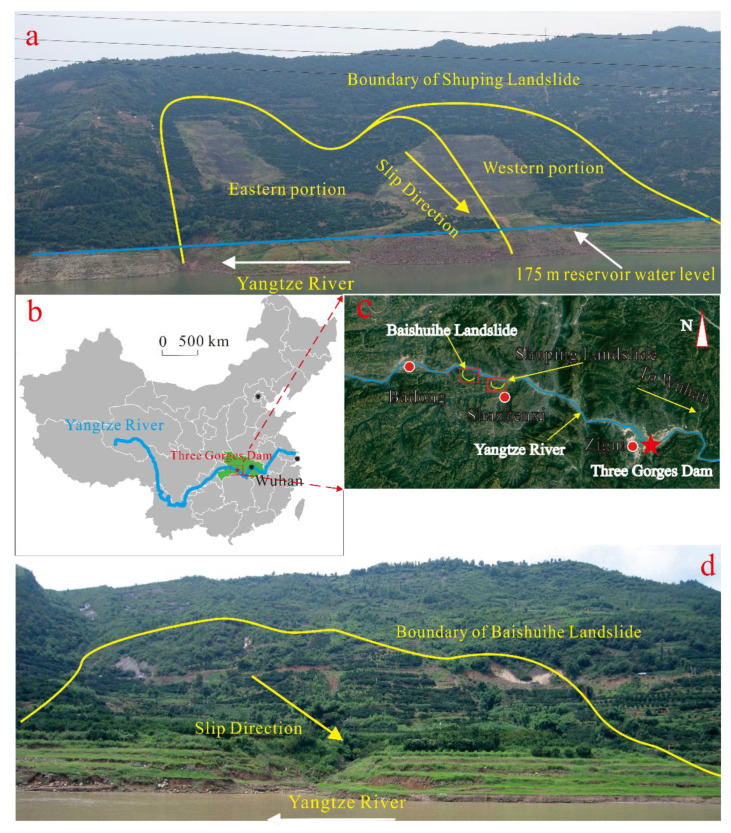
Locations of Baishuihe and Shuping landslides in the Three Gorges Reservoir area: (**a**) Shuping landslide; (**b**,**c**) location; and (**d**) Baishuihe landslide.

In this study, two giant landslides, namely the Baishuihe landslide and Shuping landslide in the Three Gorges Reservoir area (TGRA), with similar deformation characteristics, were used as study cases to develop and validate the proposed hybrid model. The Baishuihe landslide in the Three Gorges Reservoir area is located on the south bank of the Yangtze River, 56 km from the TGD and belongs to Shazhenxi town. The Shuping landslide in the TGRA is located on the right bank of the Yangtze River, 47 km away from the Three Gorges Dam (TGD), and belongs to Shazhenxi town. The locations of the two landslides are shown in Figure 2.

### 3.1. Baishuihe Landslide

#### 3.1.1. Geological Conditions

The Baishuihe landslide has a 500 m length from north to south, a 430 m width from east to west, and an approximately 30 m average thickness. With a large thick layer of soil, the sliding mass is a monoclinic stratum with sloping terrain, high in the south and low in the north, and the main sliding direction of the landslide is oriented at N 20° E. The east and west sides of landslide are bounded by bedrock ridges, with an overall slope of about 30°. The rear elevation is 450–500 m, and the front elevation is 120–130 m. The volume of the landslide is 645 × 10^4^ m^3^, covering an area of 21.5 × 10^4^ m^2^.

**Figure 3:**
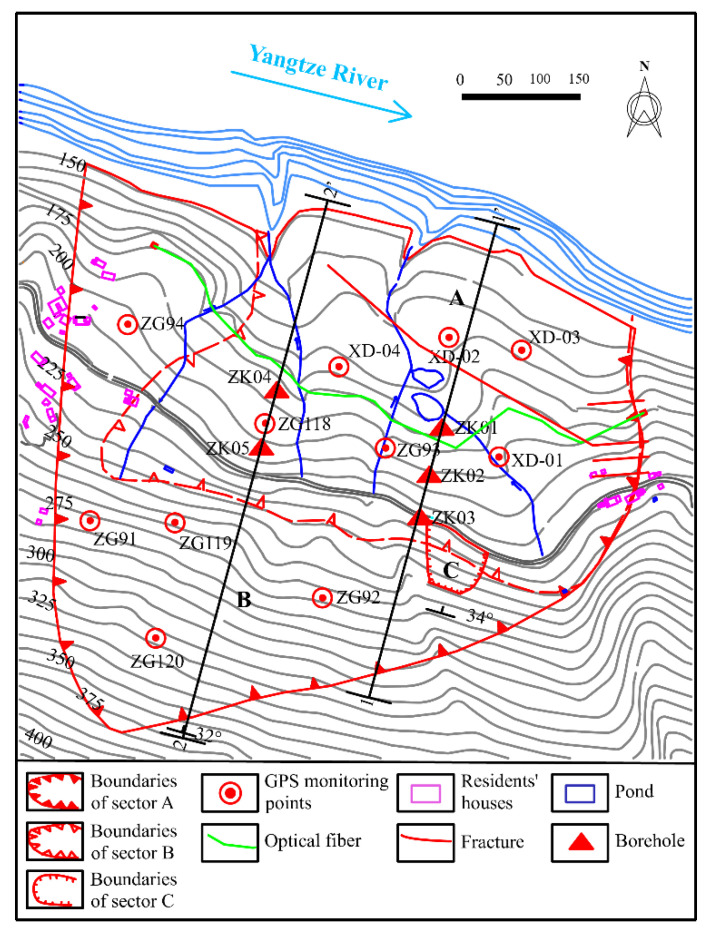
Topographical map of the Baishuihe Landslide with the location of the monitoring network.

The monitor of the Baishuihe landslide was started in June 2003, including the combination of GPS, borehole inclinometer, and distributed optical fiber. As shown in Figure 3, a total of 11 GPS monitoring sites are arranged on the surface of the sliding mass. Three borehole inclinometers were established next to the GPS monitoring sites in the middle section (profile 1 and profile 2) of the landslide and monitor synchronously with the GPS. At the same time, a distributed optical fiber across the east–west boundary of the landslide is arranged at the leading edge of the landslide for monitoring the large deformation.

**Figure 4:**
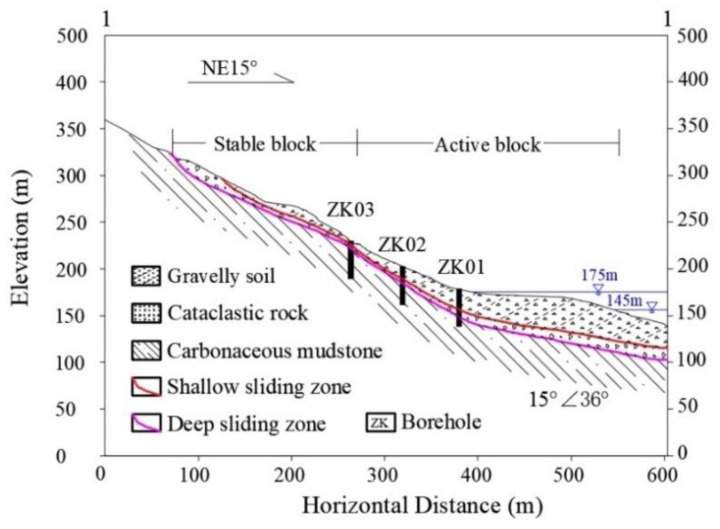
Schematic geological of profile 1.

The sliding mass of the Baishuihe landslide is mainly included in Quaternary deposits, like silty clay and fragmented rubbles with a loose and disorderly structure. The lithology of the bedrock and strata that outcrop around the landslide are medium-thick layered cataclastic rock and thin-layered carbonaceous mudstone with dip direction of 15° and dip angle of 36° (Figure 4). There are two sliding zones founded in the Baishuihe landslide with different depths. The upper one is located at the interface between Quaternary deposits and cataclastic rock, with a depth varying from 12 to 21.5 m. The lower one is located at the interface between the cataclastic rock and carbonaceous mudstone at a depth varying from 27.5 to 30 m.

#### 3.1.2. Monitoring Data and Deformation Characteristics of the Landslide

**Figure 5:**
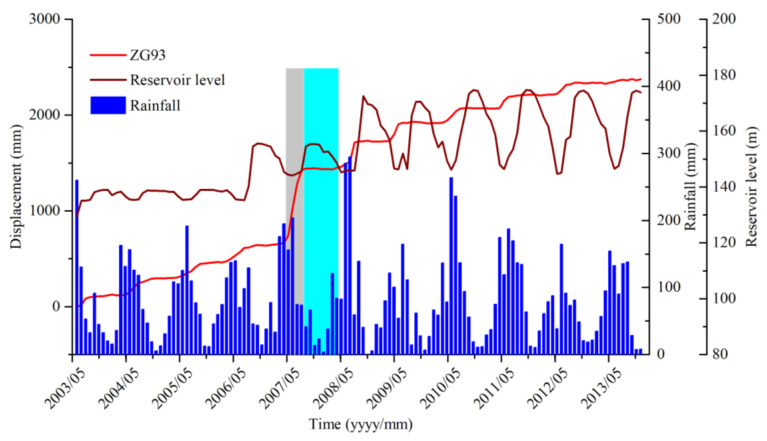
Rainfall, reservoir level, and accumulated displacement of monitoring sites ZG93 in the Baishuihe landslide.

Figure 5 shows the monitoring results of rainfall, reservoir level, and accumulated displacement of monitoring sites ZG93 in Baishuihe landslide from June 2003 to December 2013. The landslide velocity is generally highest from May to August, at the end of the period of decreasing water level. In the remaining months, the landslide displacement remains stable, which shows a strong corresponding relationship between the periodic fluctuation of the reservoir water level and accumulated displacement, in this case, the increase in water level. Besides, the landslide displacement shows a hysteresis effect, which is, the water level drops for 1–2 months before the landslide displacement begins to increase gradually, and when the water level stops falling, the landslide displacement continues for 1–2 months [37].

Besides, rainfall in Zigui County was concentrated from May to October. The results show that accumulated displacement accelerated quickly from May to October (gray shaded area) each year and remained stable in the remaining months (green shaded area), which is in good agreement with variations in the monthly rainfall intensity [22].

### 3.2. Shuping Landslide

#### 3.2.1. Geological Conditions

The Shuping landslide has an 800 m length from north to south, a 670 m width from east to west, and an approximately 10–70 m of thickness. The volume of the landslide is 2070 × 10^4^ m^3^, covering an area of 1575 × 10^4^ m^2^. The Shuping landslide located in the Zigui Basin between the mountains of western, with a topography of high in the south and low in the north. The rear elevation of the landslide is 470 m with a stepped landform, and the front elevation is 175 m. Multiple platforms are distributed on the sliding mass, with a slope of 5–35°, 22° on average. The landslide is in the subtropical monsoon climate zone, therefore, heavy rainfall that mainly concentrated in May–September in the form of the rainstorm.

**Figure 6:**
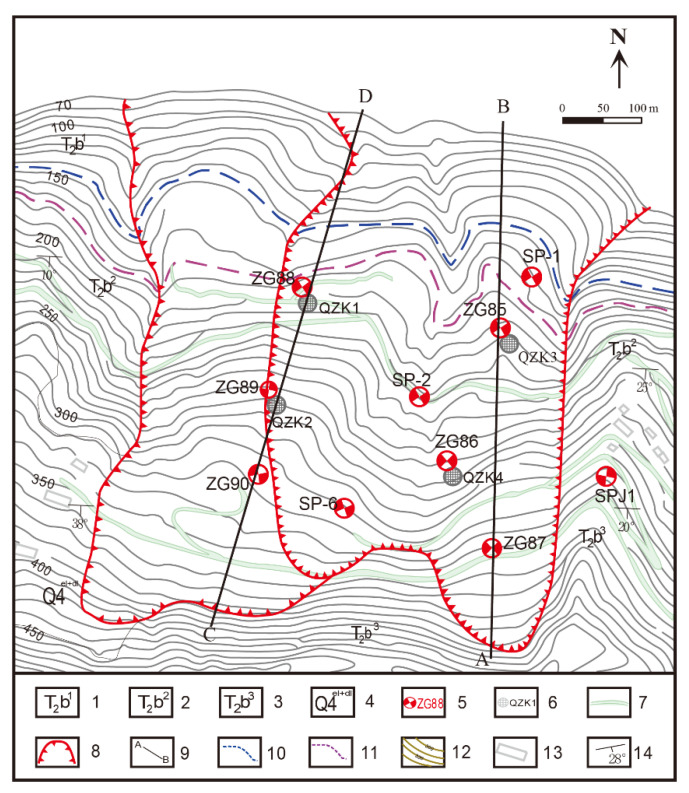
Topographical map of the Shuping landslide with the location of the monitoring network: (1) Middle Triassic Badong formation section; (1, 2) Middle Triassic Badong formation section; (2, 3) Middle Triassic Badong formation section; (3, 4) Quaternary colluviums; (5) GPS monitoring stations and number; (6) inclinometer borehole; (7) roads; (8) landslide boundary; (9) longitudinal section; (10) reservoir water level (145 m); (11) reservoir water level (175 m); (12) counter line; (13) houses; and (14) lithology orientation [25].

Shuping landslide is a large ancient landslide with poor stability, which has experienced a slip deformation many times in history. However, since the first impoundment of the TGR on 15 June 2003, significant deformation began to show up in the landslide, cracking occurred in roads and houses, especially in May to September each year. Therefore, multiple monitoring methods were adopted to measure deformation characteristics and the stability of the landslide and to observe the interactions between different portions of the landslide. The professional monitoring started in June 2003, equipped with GPS monitoring of surface displacement, tilt monitoring of deep displacement drilling, etc. A total of 10 GPS monitoring sites and 4 boreholes were arranged on the Shuping landslide, and the distribution of each monitoring site is shown in Figure 6.

#### 3.2.2. Monitoring Data and Deformation Characteristics of the Landslide

According to the results shown in Figure 7 from July 2003 to October 2013, including the rainfall, reservoir level, and accumulated displacement of monitoring sites ZG85, ZG86, and ZG87, “step-like” deformation characteristics after the first impoundment were exhibited. The displacement curves suddenly increased from May to September of each year and stabilized from October to April of the following year. The deformation characteristics displayed by GPS were very consistent with the on-site investigation of landslide’s deformation characteristics, which is, during the rise stage of displacement curves, the surface cracks increased, widened, and expanded; Progressively, the extension of the cracks on the west side showed a connection trend [25]. Additionally, the displacements in the middle (ZG86) and head scarp (ZG85) areas were greater than that in the back scarp (ZG87) area of longitudinal section A–B, which suggests that the displacements of Shuping landslide displayed retrograde-style deformation from the lower part to the upper part.

**Figure 8:**
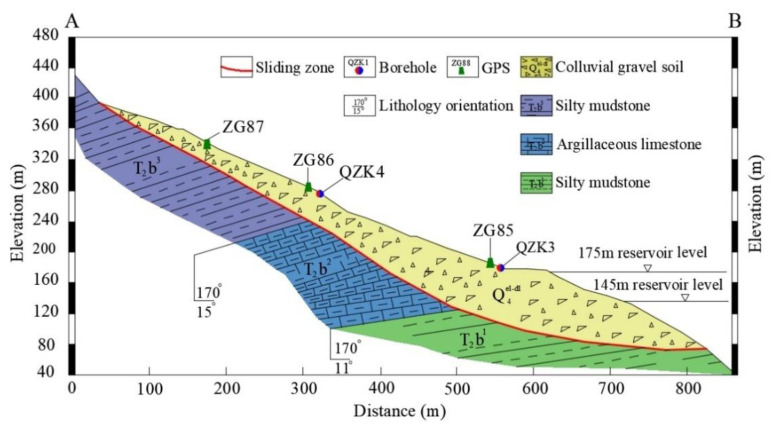
Geological cross-section of the Shuping landslide (profile A–B in Figure 6).

**Figure 9:**
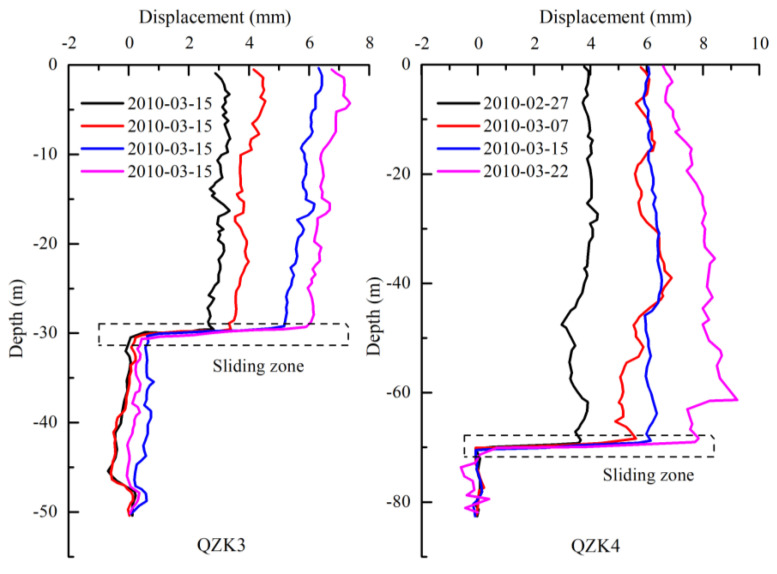
Lateral displacement versus depth of borehole QZK3 and QZK4.

Figure 8 shows two boreholes that were arranged on the Shuping landslide to monitor the deep displacement and results are shown in Figure 9. The abrupt position of the curve was the burial depth of the sliding zone, which was consistent with the sliding zone depth exposed by the exploration borehole. During the monitoring in March 2010, the rate of landslide deformation along the landslide was 0.17–0.37 mm/d, indicating that the Shuping landslide continued to creep along with the landslide. The periodic fluctuation of the reservoir water level in the TGR induced the activation of the Shuping landslide, and the rainfall accelerated the deformation of the landslide (Figure 9). The mechanism of inducing factors affecting the deformation of Shuping landslide was similar to that of the Baishuihe landslide.

### 3.3. The Selection of Inducing Factors

**Figure 10:**
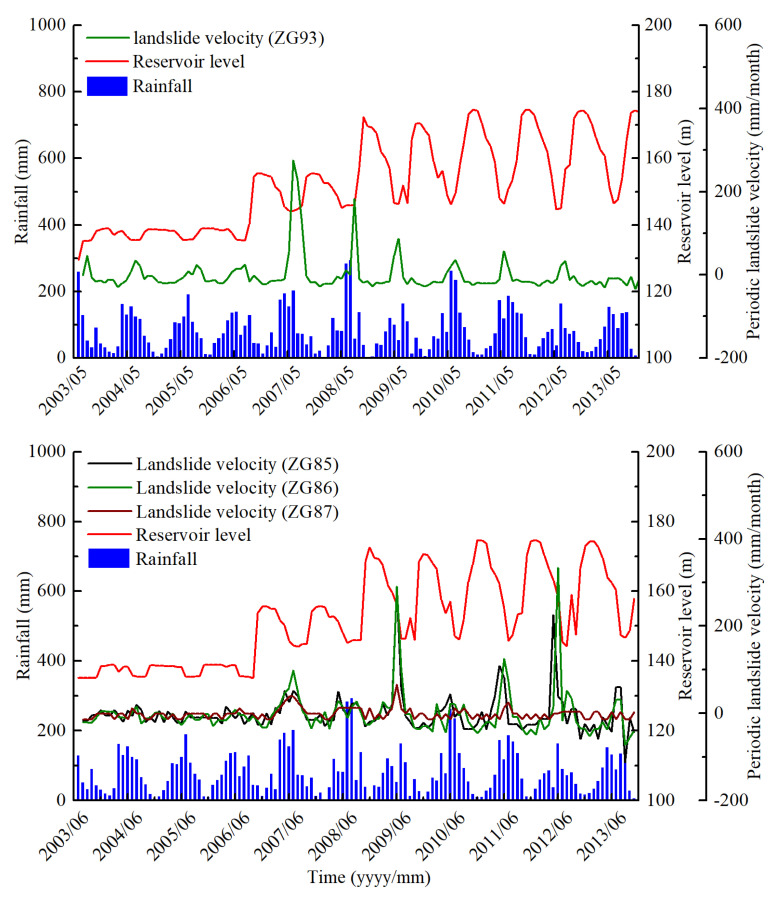
Comparison of landslide velocity, rainfall, and reservoir level of ZG93 located in the Baishuihe landslide and ZG85-87 located in the Shuping landslide.

The selection of inducing factors is critical for the prediction of the periodic component. Studies show that the displacement rate of the periodic component has a strong correlation with rainfall and reservoir level [15]. The displacement rate of the periodic component increased significantly under the influence of heavy and dense rainfall in the rainy season through two approaches. On one hand, the precipitation seeped into the rock fracture or soil porosity and raised the groundwater level in the sliding mass, which softened the rock mass or soil in the sliding zone and reduced the sliding resistance on the other hand, the continuous rainfall fully saturated the sliding mass and increased the weight of the sliding mass. As a result, the landslide displacement increased. Figure 10 shows how the displacement rate of the periodic component was positively related to rainfall, while the time of displacement rate changes shows a slight lag hysteresis compared to changes in rainfall.

The reservoir level changes also play a vital role in the affecting of landslide deformation. As is shown in Figure 10, when the reservoir level rises, the displacement rate of the periodic component will decrease accordingly. When the reservoir level is maintained stable, the displacement rate of the periodic component will tend to be gentle. Generally, the periodic fluctuation of the reservoir level will cause the fluctuation of the displacement of the periodic component.

Therefore, the influence of the reservoir level and rainfall must be considered in predicting the displacement of the periodic component. Due to their close spatial location, the rainfall and reservoir water levels of Baishuihe landslide and Shuping landslide were considered to be the same.

## 4. Results and Comparison

### 4.1. Displacement Decomposition

As mentioned above, GPS sites ZG93 on the Baishuihe landslide and GPS sites ZG85-87 on the Shuping landslide were selected to be applied and validate the proposed model:

(1) For site ZG93, the monitoring data from June 2003–October 2011 was selected as the training dataset and the remaining data from November 2011–December 2013 was used to test the model.
(2) For sites ZG85-87, the monitoring data from July 2003–October 2011 were selected as the training dataset, while the data from November 2011–November 2013 was used to test the model.

**Figure 11:**
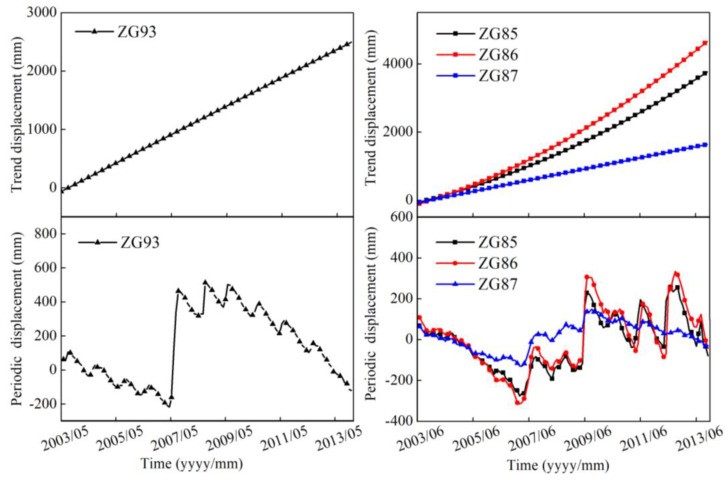
Trend and periodic components of ZG93 measurements in the Baishuihe landslide and ZG85-87 measurements in the Shuping landslide.

Through CEEMD decomposition, the trend component and a periodic component can be obtained and shown in Figure 11. In this study, the number of trials (N) was 200 and the standard deviation of the added white noise in each ensemble member (ε) was set to 0.2. After the decomposition, the displacement time series could be decomposed into several IMFs and a corresponding trend component (residue). The periodic displacements could be obtained by subtracting the trend component from the total displacement time series.

See the residue as the corresponding trend component, all of them show a steady upward trend after decomposition, which reflects the overall trend of accumulated displacement. The periodic component shows a cyclical, sharp increase–decrease trend with different amplitudes, and the first sharp upward trend occurred when the TGR was first impounded in 2007. The magnitude of the periodic component was significant and an important part of the total landslide displacements. The accuracy of periodic component prediction greatly affected the accuracy of total displacement prediction.

### 4.2. CEEMD Decomposition of Inducing Factors

The frequency component of inducing factors has normally been ignored in the existing research, but the study shows its importance [11]. In this study, after being reconstructed by the CEEMD and a *t*-test, the frequency components of the rainfall and reservoir level were used in the prediction of the periodic component.

**Figure 12:**
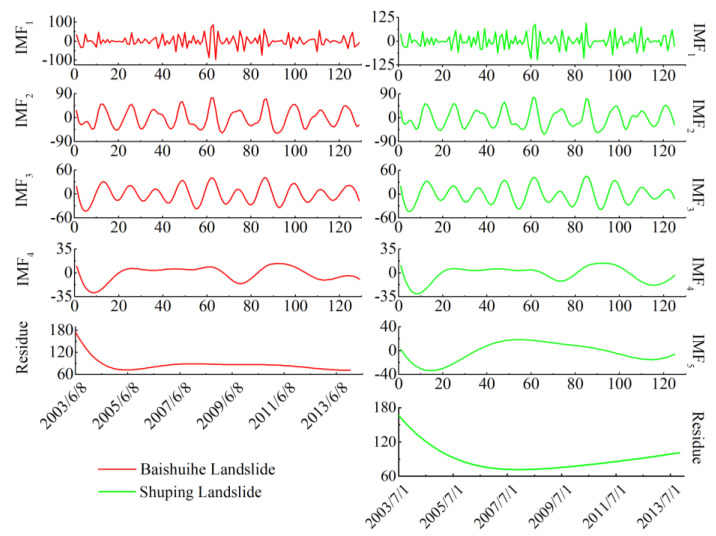
The intrinsic mode functions (IMFs) and residue of the rainfall derived through CEEMD.

**Figure 13:**
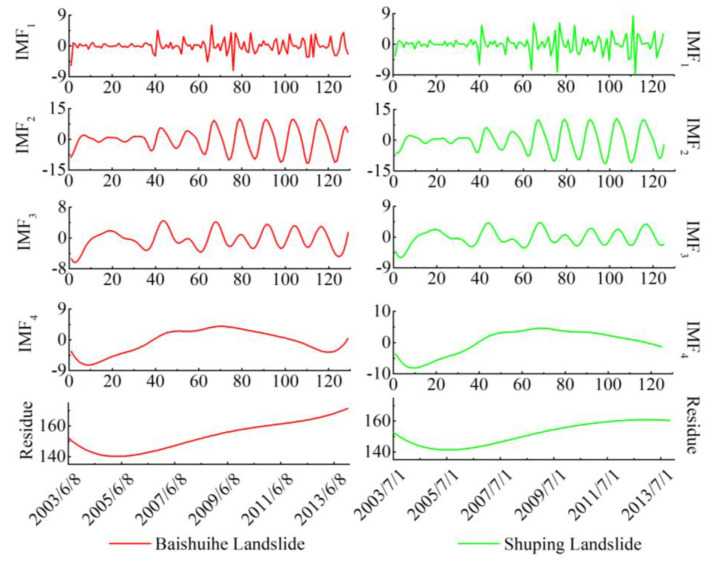
The IMFs and residue of the reservoir level derived through CEEMD.

To simplify the calculation, the date of the inducing factor was simplified to the order of the month. Set the number of trials N=200 and the standard deviation of the added white noise in each ensemble member ε=0.2. The decomposition results of the rainfall and reservoir level shown in Figure 12 and Figure 13. All of the IMFs are listed in the order of when they were extracted, which is from the highest frequency to the lowest. For the Baishuihe landslide, the rainfall time series and reservoir level time series were both decomposed into four IMFs and a residue. For the Shuping landslide, the rainfall time series was decomposed into five IMFs and a residue while the reservoir level time series was decomposed into four IMFs and a residue. The highest frequency component IMF_1_ fluctuated violently, with the largest fluctuation amplitude, while the subsequent components also show fluctuations, and the maximum amplitude of fluctuation decreased in turn. The residue of the rainfall and reservoir level is a downward concave long-period curve without any local fluctuation information, which reflects the stable trend of the rainfall and reservoir level during the monitoring period and was not used for volatility analysis.

The mean of IMF_1_ and other IMF components were calculated by a paired *t*-test with a significance set at 0.05 (2-tailed). The significance value less than 0.05 means that the difference is statistically significant. The residual component was abandoned here because it had little effect on the periodic displacement. The results in Table 1 show that significance values corresponding to the mean IMF_1_ and other IMF components of the rainfall were all greater than 0.05, indicating that the difference between the mean of other IMF components and IMF_1_ was significant. Therefore, for both landslides, the high frequency rainfall component of them was a superposition of all IMF components.

Table 2 shows the IMF’s mean value *t*-test results of the reservoir levels, the significance values corresponding to the Shuping landslide show the same pattern as that of rainfall, so the high frequency reservoir level component of it is a superposition of all its IMF components. For the Baishuihe landslide, the significance values corresponding to the mean of IMF_4_ were smaller than 0.05, indicating that the difference between the means of IMF_4_ and IMF_1_ was not significant. Therefore, the superposition of IMF_1_ to IMF_3_ was taken as the high frequency reservoir components, while IMF_4_ was used as the low- frequency time series.

**Figure 14:**
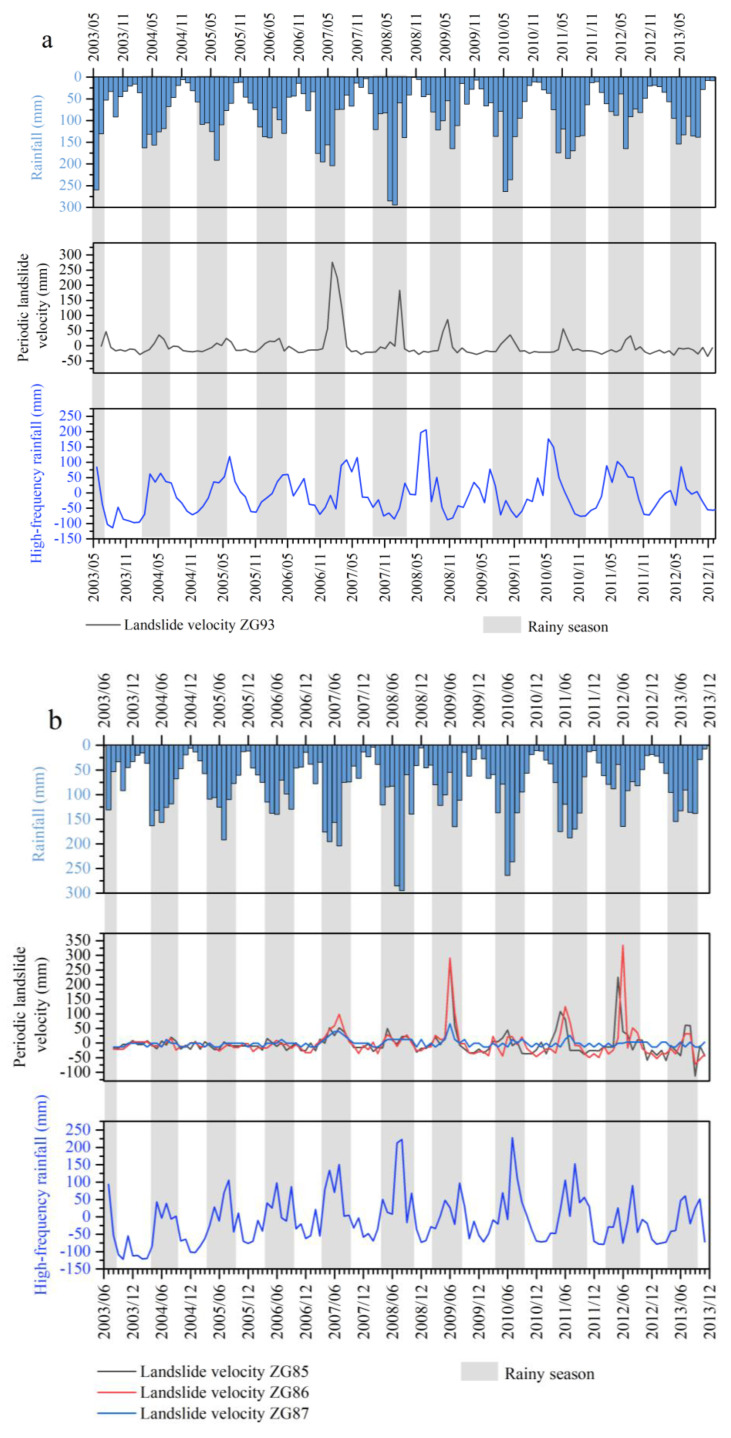
Landslide velocity compared with rainfall: (**a**) Baishuihe landslide and (**b**) Shuping landslide.

**Figure 15:**
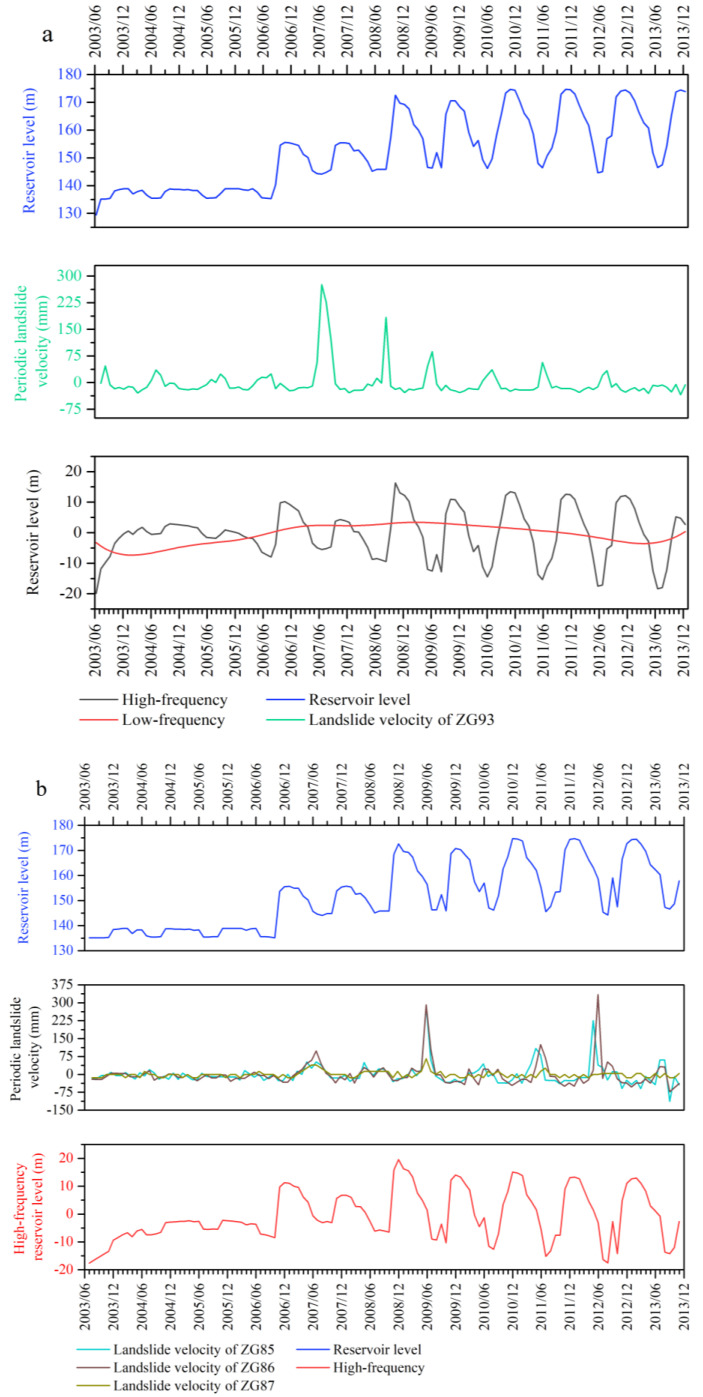
Landslide velocity compared with the rainfall reservoir level. (**a**) Baishuihe landslide and (**b**) Shuping landslide.

The obtained frequency components of rainfall and reservoir level were compared with the periodic landslide velocities in Figure 14 and Figure 15.

The comparison between the periodic rainfall and the periodic velocities in Figure 14 shows a similar pattern for the fluctuations except that there was sometimes an offset in the timing of the peaks and valleys. In the rainy season, peaks appeared in both high frequency rainfall and landslide displacement velocity curves, which illustrates that there is a strong correspondence between rainfall and landslide deformation.

The comparison between high and low frequency reservoir water levels and the periodic landslide velocities in Figure 15 shows a positive correlation between the high frequency reservoir level and the periodic landslide velocity. However, the periodic landslide velocity’s fluctuation pattern also shows a distinct lag. Besides, the low frequency reservoir level had a low correlation with the landslide movements.

### 4.3. DTW Analysis of Inducing Factors

To identify the factors that were most strongly correlated with the periodic landslide displacement, we reorganized the rainfall and reservoir level data to a different form. The rainfall data were reorganized as the current monthly rainfall (J1), the cumulative rainfall in the previous two months (J2), the cumulative rainfall in the current month and the previous month (J3), and the cumulative rainfall in the current and previous two months (J4). So, we reorganized the reservoir level data to get K1-K4, as K1 for the monthly reservoir level, K2 for the monthly change, etc. In this study, high frequency rainfall (J5), the high frequency reservoir level (K5), and the low frequency reservoir level (K6) that were reconstructed by CEEMD were used to combine with the above-mentioned factors for the prediction of the periodic landslide displacements.

The DTWD are shown in Table 3 after calculating through DTW between the inducing factors and the periodic landslide displacements at the Baishuihe landslide and Shuping landslide. The inducing factors were divided into two groups include the rainfall and reservoir level, in which, the factor with the smallest DTWD would be selected as the input variable for the SVR model. The result shows that for the Baishuihe landslide, the factor with the smallest DTWD is the current monthly rainfall (J1) in the rainfall group and the change between two months (K3) in the reservoir level group. Therefore, when predicting the Baishuihe landslide, only J1 and K3 were selected as the input variable for the prediction model. For Shuping landslide, the high frequency rainfall (J5) and the monthly change (K2) in the reservoir level group were chosen for the prediction model.

### 4.4. Predict and Comparative Analysis

#### 4.4.1. Displacement Prediction of the Trend Component

To simplify the calculation, the date of the cumulative displacement curve was simplified to the order of the month in this section. The trending component of displacement had a linear pattern of steady growth over time, which could be fitted and predicted by the least square method. A quadratic polynomial form was used as bellow:

(13) $$f\left(t\right)=at^{2}+bt+c,$$

The results are shown in Table 4.

**Figure 16:**
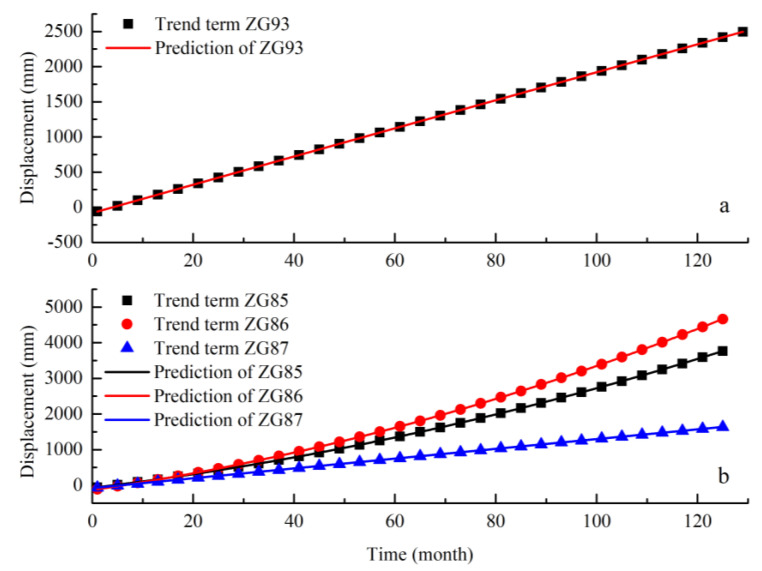
Prediction and comparison of the trend component. (**a**) Baishuihe landslide and (**b**) Shuping landslide.

The parameters of polynomial fitting in Table 4 were used to predict the trend component, and the results are shown in Figure 16. Under the polynomial fitting, the accuracy index R^2^ reached 1, and the root means square error (RMSE) was 1.670, 0.025, 0.7173, and 0.937 respectively, indicating that the results of trend component prediction were good.

#### 4.4.2. Displacement Prediction of the Periodic Component

For the establishment of the periodic component prediction model, a certain number of training samples were used to train in the SVR model, to build a regression mapping relationship between the displacement and the selected variables. Once the SVR model finished training, the output value of test samples was the periodic component of the current month.

To compare their optimized performance, the GA, ACO algorithms were used to search for the optimal penalty factor and the kernel function parameter as results are shown in Table 5. The parameter settings in the models are as follows:

(1) GA-SVR parameter setting. In sample data preprocessing, all the factors and the periodic component of displacement were normalized to the [0,1] format. In the parameter setting, the max generation default and the size of the population default are 200 and 20 respectively, and the boundary of the penalty factor and kernel function parameter is set to C=[0.1,1000] and g=[0.01,100]. The principal component is set to 95%, and the cross-validation value is v=5  [22].

(2) ACO-SVR parameter setting. In sample data preprocessing, all the factors and the periodic component of displacement were normalized to the [0,1] format. In the parameter setting, the ranges of the penalty factor *C* and kernel function parameter *g* were the same as those used above. For the ACO, the number of ants M and the maximum max times of iterations $t_{max}$ is default at 50 and 100 respectively. The evaporation rate is set to ρ=0.6.

(3) The input variables for the periodic component of ZG93 in the Baishuihe landslide are the current monthly rainfall (J1), the reservoir level change between two months (K3), and the periodic displacement component for the previous month. The input variables for the periodic component of ZG85, ZG86, and ZG87 in the Shuping landslide are the high frequency rainfall (J5), the reservoir level monthly change (K2), and the periodic displacement of the previous month.

**Figure 17:**
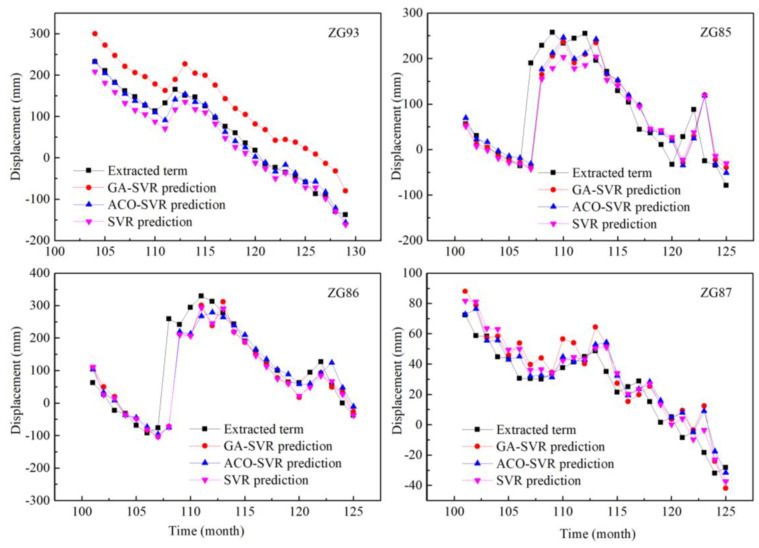
Prediction and comparison of periodic displacement of ZG93 located in the Baishuihe landslide and ZG85-87 located in the Shuping landslide.

The error of each model and its prediction accuracy are shown in Table 6 and compared in Figure 17.

Overall, the predicted periodic displacements through the proposed hybrid prediction model matched well with the observed displacements, usually with the largest R^2^ value and smallest RMSE value among the given models.

#### 4.4.3. Predicted Cumulative Landslide Displacement

**Figure 18:**
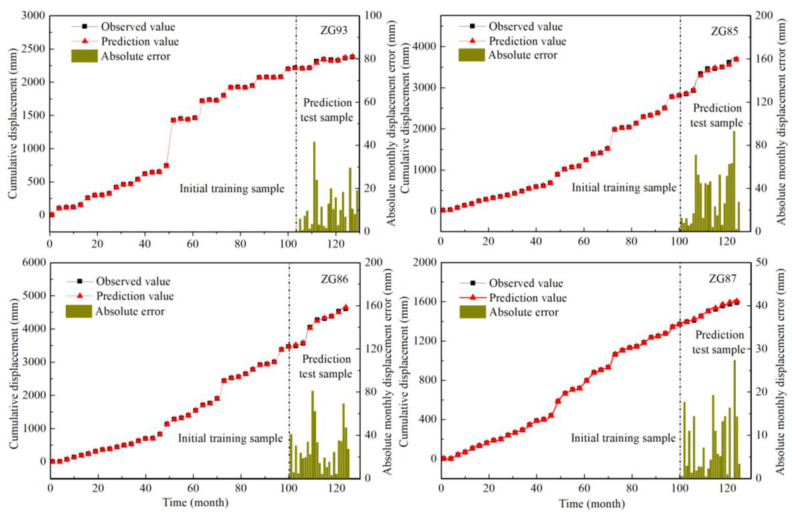
Comparison of observed and predicted accumulated displacement of ZG93 located in the Baishuihe landslide and ZG85-87 located in the Shuping landslide.

The proposed hybrid prediction model could also be used to predict the cumulative displacements. In this approach, the trend and the periodic landslide displacements were predicted independently and then added together to get the cumulative displacements. The observed and predicted results of ZG93, ZG85, ZG86, and ZG87 are shown in Figure 18.

According to Figure 17, a comparison of the observed and predicted displacements at these four monitoring locations shows that the curve of the predicted displacement corresponded well to the curve of the observed displacement. The predicted value was suitable for the initial sample data and could well predict displacements up to 26 months. In the prediction stage, when the displacement fluctuated sharply, the displacement prediction error was large, and when the displacement increased steadily, the displacement prediction error was small. Overall, the absolute error shows that the result had high accuracy in the short-component prediction of landslides with staged behavioral displacement.

## 5. Discussion

The landslide displacement prediction is a hot issue, owing to the long-term risks and challenges in many places of the world. It is a key step to predict and mitigate future landslides. However, due to the nonlinear characteristics of the landslide displacement dataset, the accurate prediction of the landslide occurrence needs a lot of resources and is difficult to implement. Although various methods have been proposed to predict the landslide displacement, the prediction accuracy of these methods is still controversial and uncertainty [9]. Actually, the high degree of uncertainty in the landslide displacement prediction makes it difficult for any single or specific model to be considered as the most suitable model for all scenarios.

Accuracy prediction of the landslide displacement needs to master the control factors of landslide occurrence [49]. Traditionally, the control factors should include the factors that make the ground surface vulnerable to damage, such as the internal characteristics of underground conditions, and the factors that trigger the landslide occurrence, such as external events inducing instability. Especially, many landslides located in the TGRA have a typically “step-like” deformation behavior especially cases discussed in this study. The deformation characteristic is mainly controlled by both internal inducing factors and external inducing factors. The internal inducing factors are of significant affection to the general trend of its deformation and development. The external inducing factors such as seasonal rainfall and changes of the reservoir level directly accelerate the deformation and damage of the landslide, which is the main reason for the “step-like” development and evolution of the landslide [22]. A wide range of topographic, lithologic, seismic, hydrological, and meteorological factors should be utilized to generate a catalog of the control factors for predicting the landslide displacement. The availability of data related to the landslide displacement prediction is often the key factor to determine the selection of the control factors. Concretely, for the two Three Gorges landslides analyzed, rainfall and changers of the reservoir water level as the two main inducing factors play a vital role in the deformation analysis. Obviously, these two factors are not enough to predict the landslide occurrence. Although the inducing factors like rainfall show a significant periodic characteristic, most of the existing research has often ignored the frequency component of it and how it affects the deformation of the landslide. Most of the classical optimized SVR methods, such as PSO-SVR and GA-SVR, do landslide displacement prediction without taking the frequency component of inducing factors into consideration. Therefore, it is inaccurate to predict the deformation of landslide without consideration of the external inducing factors’ frequency component [11]. On the whole, there is no unified guideline or global standard for the accurate selection of the control factors and the number of factors that should be input into the prediction model. No single or specific model is best suited for all scenarios of the landslide displacement prediction, due to the limitation of the selection of the prediction model that depends on the potential goals, scientific objectives, model limitations, and warnings. Therefore, owing to the nonlinear mechanism of the landslide deformation, some prediction models are more attractive.

This study is committed to further improving the accuracy of the prediction model and has achieved two aspects of improvement. Firstly, CEEMD and DTW are used to extract frequency components, and the mapping relationship between independent variables and variables is established based on frequency components, which is not available in the aforementioned standard SVR model. Compared with other time-series decomposition methods, the CEEMD method has the advantage of avoiding an estimate of the function type of the trend component in advance. Through CEEMD and a *t*-test, the external inducing factors can be reconstructed into the high frequency, low frequency, and residue components. The high frequency components can reflect local information of inducing factors time series, which shows a strong correspondence with the periodic displacement velocity. The low frequency components, along with the residue components are relatively smooth and very different from the periodic displacement velocity curve, which can be ignored. Secondly, the ACO algorithm is used to further optimize the SVR model, and a hybrid prediction method composed of the ACO-SVR model is formed to realize a better selection of input variables and optimization of the SVR model. Compared with GS, GA, and PSO algorithms, the ACO method can avoid falling into a local optimum in high-dimensional space and has a low convergence rate in the iterative process. The ACO-based SVR model has a better generalization performance and can increase the predictive accuracy of landslide displacement by determining the optimal parameters of SVR automatically.

The deformation characteristic of the reservoir landslide is mainly controlled by both internal inducing factors and external inducing factors. The external inducing factors such as seasonal rainfall and changes of the reservoir level directly accelerate the deformation and damage of the landslide, which is the main reason for the “step-like” deformation and evolution of the landslide [50]. The external inducing factors show a significant seasonal characteristic that consists of frequency components. The proposed method extracts these frequency components, which can better reveal the corresponding relationship between external inducing factors and landslide deformation. Using them as input variables to be selected can help to improve the prediction effect. Therefore, the proposed method has wide applicability in the prediction of the reservoir landslide, especially suitable for landslide deformation caused by inducing factors like rainfall and reservoir water level fluctuation.

In the study, only several monitoring points were modeled, and only one hundred sets of data are very small for the proposed method. At the same time, these datasets are short for the length of the time series. When large datasets can be used to compute for the computational intelligence algorithms, the influence of controlling factors on the landslide displacement prediction can be reflected more comprehensively.

The proposed method is a data-driven model that is also known as the black-box model with a drawback of only prediction error provided and no information regarding the associated predictive uncertainties. The output of most existing data-driven models is a single estimate of each prediction range, and these single estimates that provide deterministic results are called point predictions [9]. The uncertainties can affect the accuracy of point estimates and are consisting mainly of parameter uncertainty, model uncertainty, and input uncertainty that could be substantial. Therefore, to improve the reliability and credibility of the proposed hybrid model, incorporate prediction uncertainty into point prediction to quantify the uncertainty is necessary [51].

Although the proposed method has a small absolute error and an ideal prediction result during the initial sample training stage and the displacement with steady growth, the error of predicted displacement will inevitably increase where the observed displacement changes sharply. Therefore, to establish a more accurate causal relationship, the latest monitoring data should be gradually replaced, and earlier information should be deleted [52].

## 6. Conclusions

The conclusions were summarized from this study as below:

(1) The results of the proposed hybrid model show that after considering the frequency components of landslide-induced factors, the prediction accuracy of the displacement prediction model based on ACO-SVR was better than that of other models based on SVR and GA-SVR.

(2) CEEMD with high decomposition accuracy and high operation efficiency could better highlight the local fluctuation characteristics of the inducing factors time series. It is suitable for extracting the trend component displacements of landslide’s displacements. After CEEMD decomposition, the frequency components of the landslide inducing factors could be divided into three parts, the high frequency components, the low frequency components, and a residue. The residue reflected the long-component trend of the inducing factor, and the high frequency component strongly affected the landslide periodic displacement.

(3) Overall, through the comparative analysis and prediction applied in the Baishuihe landslide and Shuping landslide, it was proved that the proposed hybrid displacement prediction method based on CEEMD reconstruction and DTW-ACO-SVR model was effective. This method has the potential for broad application to predict the landslide affected by seasonal reservoir water levels and rainfall.

## Figures and Tables

**Figure 7:**
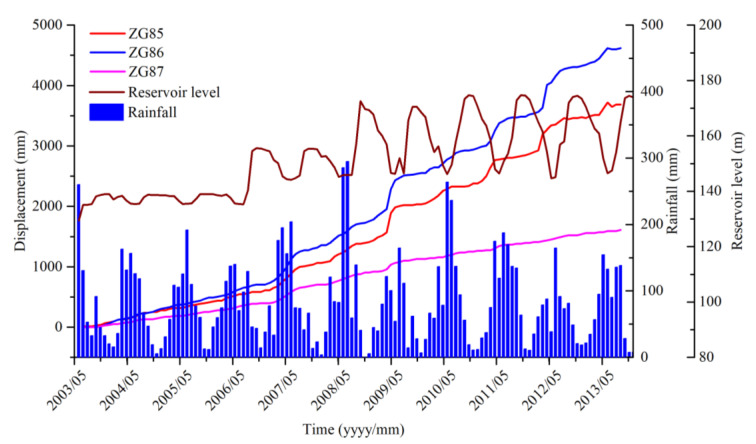
Rainfall, reservoir level, and accumulated displacement of monitoring sites ZG85, ZG86, and ZG87 in the Shuping landslide.

**Table 1:** IMF’s mean value *t*-test results of rainfall.

Landslide	Component	*t*	Significance	Mean (mm)	Standard Deviation (mm)
Baishuihe	IMF2	0.566	0.572	2.032	40.759
	IMF3	0.130	0.897	0.389	33.889
	IMF4	0.573	0.567	1.530	30.296
Shuping	IMF2	0.370	0.712	1.536	46.461
	IMF3	0.181	0.856	0.672	41.428
	IMF4	0.608	0.544	2.071	38.0096
	IMF5	0.823	0.412	2.918	39.668

**Table 2:** IMF’s mean value *t*-test results of the reservoir levels.

Landslide	Component	*t*	Significance	Mean (mm)	Standard Deviation (mm)
Baishuihe	IMF2	0.194	0.847	0.0905	5.306
	IMF3	1.082	0.281	0.265	2.783
	IMF4	2.003	0.047	0.654	3.706
Shuping	IMF2	0.223	0.824	0.110	5.537
	IMF3	0.576	0.566	0.164	3.178
	IMF4	0.400	0.690	0.168	4.693

**Table 3:** Dynamic time warping distance (DTWD) between inducing factors and periodic displacement.

Inducing Factor	Component	Baishuihe Landslide	Shuping Landslide
Rainfall	J1	14,882.36	13,389.61
	J2	17,883.66	17,619.62
	J3	17,680.98	17,712.31
	J4	20,361.35	23,160.85
	J5	16,274.97	9130.15
Reservoir level	K1	23,980.90	21,671.89
	K2	25,228.01	12,531.37
	K3	15,901.48	19,486.31
	K4	25,516.75	12,831.98
	K5	23,980.90	21,671.89
	K6	19,980.20	/

**Table 4:** Parameters of the trend component of displacement based on polynomial fitting.

Landslide	Site	a	b	c	R^2^	RMSE
Baishuihe	ZG93	0	19.98	−78.35	1	1.670
Shuping	ZG85	0.11	16.97	−71.76	1	0.025
	ZG86	0.14	20.45	−130.52	1	0.717
	ZG87	0	13.72	−76.04	1	0.937

**Table 5:** Optimal parameters of SVR used for the dataset.

Landslide	Site	GA-SVR		SVR		ACO-SVR	
		C	g	C	g	C	g
Baishuihe	ZG93	15.6388	18.6253	6.3255	0.5237	15.2037	0.0151
Shuping	ZG85	16.3740	0.0601	0.0256	0.8526	96.3522	0.0256
	ZG86	11.1647	0.5360	9.3256	0.8526	96.3522	0.0256
	ZG87	5.1747	0.2136	9.3256	0.8526	96.3522	0.0256

**Table 6:** Prediction accuracy of each model.

Model	ZG93		ZG85		ZG86		ZG87	
	R^2^	RMSE	R^2^	RMSE	R^2^	RMSE	R^2^	RMSE
GA-SVR	0.655	69.93	0.772	64.38	0.802	74.77	0.841	13.84
SVR	0.951	68.80	0.752	67.16	0.804	74.40	0.907	10.59
ACO-SVR	0.985	14.56	0.787	62.24	0.801	74.64	0.903	10.79
